# Analysis of Calving Cow Posture Recognition, Behavioral Changes, and Influencing Factors Based on Machine Vision

**DOI:** 10.3390/ani15091201

**Published:** 2025-04-23

**Authors:** Yuning An, Yifeng Song, Hehao Jiang, Yuan Wang, Na Liu, Xia Li, Zhalaga Zhang, Xiaoping An

**Affiliations:** 1 College of Animal Science, Inner Mongolia Agricultural University, Hohhot 010018, Chinawangyuan@imau.edu.cn (Y.W.); liuna1988@imau.edu.cn (N.L.);; 2 National Center of Technology Innovation for Dairy-Breeding and Production Research Subcenter, Hohhot 010018, China; 3 Key Laboratory of Smart Animal Husbandry at Universities of Inner Mongolia Autonomous Region, Integrated Research Platform of Smart Animal Husbandry at Universities of Inner Mongolia, Inner Mongolia Herbivorous Livestock Feed Engineering Technology Research Center, Hohhot 010018, China

**Keywords:** calving dairy cow, posture recognition, YOLOv8, behavioral analysis, intelligent farm management

## Abstract

This study introduces a non-contact, single-target method for real-time monitoring of dairy cow calving posture and behavior using the YOLOv8 model. In total, 600 videos were collected, from which 10,544 image samples were extracted through frame-by-frame processing. Complete video recordings of 86 cows (30 primiparous and 56 multiparous) were utilized to investigate changes in calving behavior. The YOLOv8 model achieved excellent performance with precision (*P*), recall (*R*), and mean average precision (m*AP*) of 96.72%, 96.53%, and 97.41%, respectively, and recognition *P* of 89.19% for lying postures and 82.61% for standing postures. Behavioral analysis revealed that lying postures were more frequent than standing, and primiparous cows had more frequent posture transitions (9.07 changes) than multiparous cows (5.29 changes), particularly during early parturition. Primiparous cows also showed significantly higher average times for parturition and lying as well ashigher frequency of behavioral changes compared to multiparous cows. Additionally, calf birth weight was positively correlated with maternal behaviors, especially in primiparous cows. Our proposed model effectively and accurately recognizes calving postures in dairy cows, enabling the early detection of abnormal calving events. This provides a scientific basis and technical support for intelligent farm management.

## Simple Summary

Real-time monitoring of calving posture and behavior in dairy cows is crucial for accurately assessing the calving process and ensuring precise periparturient management. This study presents a non-contact, single-target calving posture recognition method based on the YOLOv8 model for real-time monitoring of dairy cow behavior during parturition. By analyzing 600 videos from 10,544 image samples, we observed key behavioral differences between primiparous and multiparous cows. The model effectively recognizes calving postures and offers technical support for farm management.

## 1. Introduction

Calving management is a crucial aspect of modern dairy farm operations. The level of management not only directly affects herd expansion and calf survival rates but also has profound implications for production efficiency and cow welfare. Calving is a critical phase in the dairy cow’s production cycle, typically comprising three distinct stages: cervical dilation, fetal expulsion, and placental delivery. Among these, the fetal expulsion stage is particularly critical and hazardous, often leading to dystocia if labor is prolonged, adversely affecting both dam and calf [1]. During calving, cows exhibit significant behavioral and physiological changes, including elevated body temperature, reduced resting time, and heightened restlessness characterized by behaviors such as increased walking or mounting activity [2,3,4]. Additionally, variations in calving posture, such as standing versus lying down, are critical indicators of the cow’s health status, comfort level, and overall welfare [5,6]. Research has shown that lying time before and after calving is closely associated with the cow’s health condition, production performance, and well-being. For instance, longer lying times may correlate with higher milk yields and are associated with factors such as body condition score and lameness severity [7,8]. Therefore, accurately identifying and classifying these behaviors is essential for monitoring the calving process and ensuring timely interventions [9,10]. The YOLO model offers a robust method for detecting and classifying standing and lying behaviors during calving with high precision [11]. By leveraging this approach, we aim to enhance the understanding of behavioral patterns during parturition and provide valuable insights into improving animal welfare.

Despite the critical importance of calving for both cows and newborn calves, current monitoring practices in dairy farming predominantly rely on manual observation. This approach is time-consuming, labor-intensive, and often lacks accuracy, leading to delays in identifying calving difficulties [12,13,14]. As large-scale farming operations continue to expand, there is an increasing demand for more efficient and precise management strategies. In recent years, advancements in computer vision technology have introduced new possibilities through intelligent monitoring systems based on machine vision and deep learning algorithms. These technologies enable real-time monitoring of behavioral changes in cows, such as head posture and standing or lying patterns, significantly enhancing the informatization, digitization, and intelligence of farm management. This not only aids caretakers in timely intervention during the calving process but also effectively reduces the risk of dystocia, improving health and welfare of both cows and calves while boosting farm economic returns [15,16]. As a case in point, by automatically detecting physiological indicators, such as tail movement, rumination time, and body temperature, it is possible to predict imminent calving events, thereby reducing the need for manual observation and increasing monitoring efficiency [17,18,19]. Also, deep learning algorithms like the YOLO series models have been employed to detect cow eye regions with an average accuracy of 96.88% [20]. Other studies have combined temporal feature extraction networks to accurately identify various behaviors such as drinking, rumination, and walking with an average accuracy of 97.6% [21].

In this study, we employed computer vision analysis and the YOLOv8 model to identify calving postures in cows. We obtained 600 videos and extracted 10,544 image samples through frame-by-frame processing, with a total duration of 3085 min, to construct a model for recognizing calving postures. Complete videos of 86 cows (30 primiparous and 56 multiparous) were utilized to investigate changes in calving behavior. Our developed dataset allows for precise identification of key postures during the calving process and further analyzes the characteristics of posture behavior changes. Additionally, through correlation analysis, we explored the main factors influencing calving behavior. This research provides scientific evidence and technical support for intelligent farm management.

## 2. Materials and Methods

### 2.1. Experimental Design

The experiment was conducted from March 2023 to July 2024 at a large dairy cattle farm located in the suburban area of Hohhot (Hohhot, Inner Mongolia, China, 40°48′ N, 111°39′ E). Healthy Holstein dairy cows with a body condition score (BCS) ranging from 3.25 to 3.50 were selected as study subjects. As the calving date approached and the cows exhibited signs of parturition, they were transferred from the periparturient pen to the calving pen (dimensions: 10 m × 10 m) and entered the calving management phase. Subsequently, the calving process was monitored utilizing cameras to capture video footage, which was then used to develop and independently validate a calving posture recognition model. Our model development included a total of 86 calving dairy cows, comprising 30 primiparous cows and 56 multiparous cows. All cows with abnormal calving, including those experiencing dystocia, were excluded from the study.

### 2.2. Video Capture

**Figure 1:**
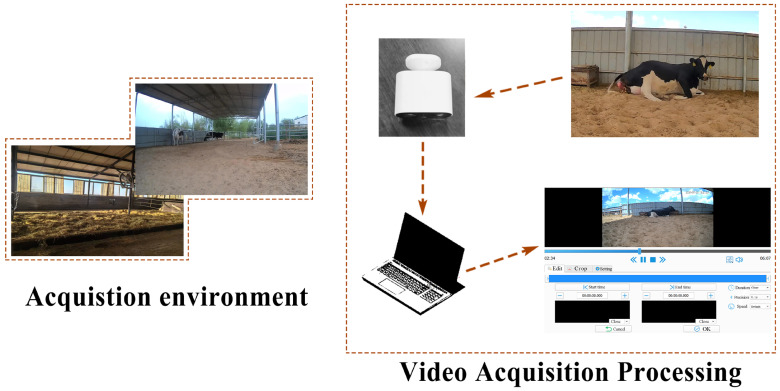
Schematic Diagram of video collection and processing.

For the purpose of facilitating real-time monitoring of dairy cows during parturition, we implemented a continuous video surveillance system within the calving pens. High-definition infrared from Imou (model K22-4M-D; Hangzhou Huacheng Network Technology Co., Ltd., Hangzhou, China) was strategically installed to ensure comprehensive coverage and unobstructed views of the cows. The camera operated continuously and was connected to a smartphone, enabling real-time observation of the cows’ parturition status. The recorded videos were periodically transferred to an external hard drive for storage. Cameras were strategically positioned around the cows’ birthing pens, with each camera placed approximately 4 m apart and at a height of about 1 m. A total of five cameras were used, with each camera dedicated to filming one cow in labor at a time (Figure 1). The dairy farm operates under the following lighting schedule: from 8:00 pm to 6:00 am during spring, summer, and autumn, and from 6:00 pm to 7:00 am in winter. Each cow pen is equipped with five large lamps installed at a height of 4.5 m. During image acquisition, recordings were made under natural light during the day, whereas at night, auxiliary LED lighting was used. The LED lamps were sourced from Guangdong Zhongshan Ant Lighting Optoelectronics Co., Ltd., Zhongshan, China (specifications: 5000 V, 100 W).

To monitor the calving process in dairy cows, we collected 600 video recordings of calving events. These videos were utilized to develop a YOLOv8-based recognition model for identifying calving postures in dairy cows. After excluding images without cows, the videos were processed at a frame rate of 10 frames per second (fps) to extract individual frames, which were then used to construct an image dataset of calving postures. This sampling rate was determined based on preliminary observations that the shortest duration for a cow transitioning from a lying to a standing posture was approximately 0.8 min. Additional videos were captured under various conditions, encompassing daytime, nighttime, and rainy weather, focusing on different body parts such as head, tail, and abdomen. Images for an independent validation dataset were acquired at a frame rate of 1 frame per minute (fpm). The complete birthing process of 86 cows (comprising 30 primiparous and 56 multiparous cows) was fully monitored. Videos documenting the entire birthing process were processed, and the birth weights of the calves were recorded. Given that the shortest duration for a cow to transition from lying down to standing up in our video data were 0.8 min, a frame rate of 2 frames per minute (fpm) was used for analyzing changes in bovine birthing posture behavior.

### 2.3. Image Data Collection, Annotation and Augmentation

**Figure 2:**
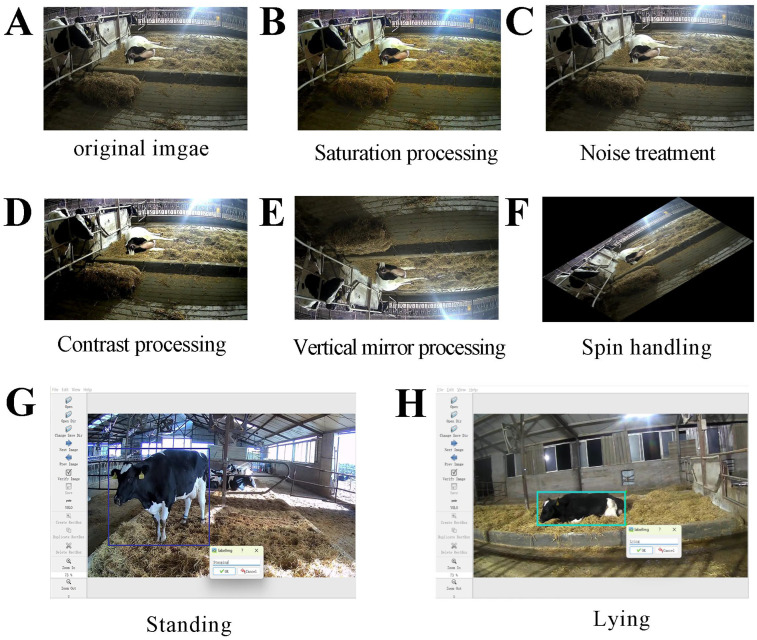
Image augmentation techniques and LabelImg annotation during the calving process in dairy cows. (**A**) Original image, (**B**) saturation adjustment, (**C**) noise addition, (**D**) contrast adjustment, (**E**) vertical mirroring, (**F**) rotation, (**G**) standing posture annotation, (**H**) lying posture annotation.

Video footage capturing dairy cows during parturition was recorded from various angles. Images were extracted from these videos at a resolution of 2304 × 1296 pixels and saved in JPG format. For data cleaning, we removed images that were either highly similar to each other or blurry. The selected images were then subjected to data augmentation techniques such as saturation enhancement, noise addition, contrast adjustment, vertical mirroring, and rotation. These processes increased our dataset to a total of 10,544 effective images (Figure 2A–F). All image datasets were uniformly stored as TXT files for subsequent model training and validation.

During parturition, dairy cow postures were observed to be either standing or lying (Table 1). These postures were annotated accordingly operating the “LabelImg” tool within a Python (v 3.9.0) environment (Figure 2G,H). Eventually, the annotated images were split into training, testing, and validation sets with an 8:1:1 ratio.

### 2.4. Application of YOLOv8 for Object Detection and Posture Recognition

**Figure 3:**
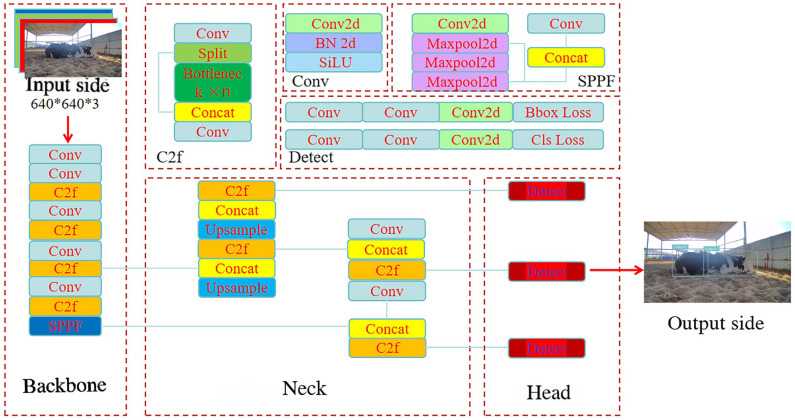
Schematic representation of the YOLOv8 architecture.

YOLOv8 is a deep learning-based object detection algorithm designed for object recognition and localization [22] (Figure 3). The images from previously constructed training set were resized to a fixed resolution of 640 × 640 pixels and then fed into the YOLOv8 model. Through systematic processing, the model achieved favorable training outcomes, enhancing its robustness and accuracy.

The performance of the experimental model was evaluated using several key metrics: precision (*P*), recall (*R*), average precision (*AP*), mean average precision (m*AP*), and *F*_1_ score. The calculation formulas are as follows:

(1) $$P=\frac{T_{p}}{T_{p}+F_{p}}\times 100\%$$

(2) $$R=\frac{T_{p}}{T_{p}+F_{n}}\times 100\%$$

(3) $$F_{1}=\frac{2\times PR}{P+R}$$

(4) $$AP=\frac{\sum_{k=1}^{n}P(K)\Delta r(K)}{R}$$

Here, *T_P_* refers to the number of correctly classified samples; *F_P_* represents to the number of incorrectly classified samples; and *F_N_* denotes to the number of samples that were misclassified as incorrect despite being correct. Additionally, *C* refers to the total number of label types.

Additionally, an independent validation image set was constructed. These images were annotated following the same procedure described earlier. Thereafter, the previously trained YOLOv8 posture recognition model was applied to this independent validation set, with m*AP* serving as the primary evaluation metric for assessing model performance.

### 2.5. Behavioral and Postural Analysis of Dairy Cows During the Calving Process

After establishing the YOLOv8 model, we further applied it to analyze the complete calving process of 86 dairy cows, identifying their postures throughout parturition. This analysis yielded the number of posture-specific images and calculated the posture indices during calving, which include number of lying events (*NL*), number of standing events (*NS*), and number of posture changes (NPC) (transitions between lying and standing postures). The overall posture index for the calving process (number of parturitions, *NP*) was computed leveraging the following formula:

(5) NP=NL+NS

Moreover, we calculated duration metrics associated with the calving process and closely observed three distinct phases of parturition in dairy cows: the cervical dilation phase (early stage), the fetal expulsion phase (mid stage), and the placental expulsion phase (last stage). These metrics included the time of parturition (*TP*), time of lying (*TL*), time of standing (*TS*), and frequency of behavioral changes (*FBC*). The total behavioral time during calving was calculated as the sum of posture durations applying the following formula as:

(6) TP=TL+TS

(7) $$TP=NP\times \frac{30s}{frame}$$

(8) $$TL=NL\times \frac{30s}{frame}$$

(9) $$TS=NS\times \frac{30s}{frame}$$

The FBC was defined as NPC during parturition. Specifically, this corresponds to the number of occurrences of standing behavior during the calving process. It was calculated employing the formula:

(10) $$FBC=\frac{NPC}{2}$$

### 2.6. Statistical Analysis

The experimental environment and equipment were detailed in Table 2. Statistical analyses of the data were performed using Excel 2022. Additionally, we employed Origin 2022a software to perform correlation analysis and generate plots. We adopted a two-tailed test, where a *p*-value of less than 0.05 was taken as evidence of a significant difference. To compare differences between two sets of data, an Independent Samples *t*-test was implemented. For comparisons among three or more groups, a One-Way ANOVA was carried out. This analysis helped us evaluate whether significant differences existed in the means across different groups. If the ANOVA results suggested significant inter-group differences, post hoc testing, specifically Tukey’s HSD test, was conducted to identify the pairs of groups with significant disparities. Prior to conducting the *t*-test and ANOVA, normality tests were essential to ensure that the statistical assumptions were met. Specifically, we utilized the Shapiro–Wilk test and Kolmogorov–Smirnov test to assess whether the data conformed to a normal distribution. Pearson correlation analysis was employed to evaluate relationships between variables, with a significance threshold set at *p* < 0.05.

## 3. Results

### 3.1. Dataset Construction and Analysis of Postural Behavior in Dairy Cows During Calving Process

In our study, we constructed a dataset leveraging 600 video recordings of dairy cows during calving, with a total duration of 3085 min. From these videos, we extracted

**Figure 4:**
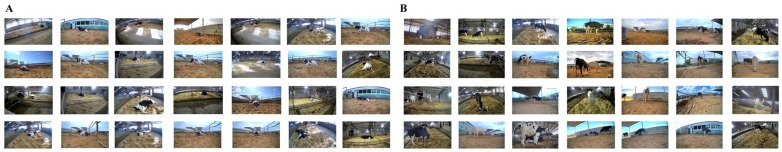
Examples of posture images of dairy cows during parturition. (**A**) Standing up and (**B**) lying down.

10,544 image samples through frame-by-frame processing. Among these, 4548 images depicted standing postures, while 5996 images represented lying postures. The dataset was divided into training, testing, and validation sets for the YOLOv8 model in an 8:1:1 ratio. Specifically, the training set included 8436 images, while both the testing and validation sets consisted of 1054 images each (Figure 4). In addition to this primary dataset, we also constructed an independent validation dataset comprising a total of 1805 images captured under various conditions. This dataset included 360 daytime images, 382 nighttime images, and 185 rainy-day images. Additionally, it was categorized into region-specific samples, consisting of 287 head-focused images, 266 tail-focused images, and 325 abdomen-focused images.

In an effort to study the patterns of postural behavior changes during the complete calving process, we analyzed video recordings from 86 dairy cows (Table 3). This included videos from two groups: primiparous cows (*n* = 30) with a total video duration of 1257 min and multiparous cows (*n* = 56) with a total video duration of 1547 min. The average birth weights of newborn calves were recorded as 37.32 kg for primiparous cows and 38.96 kg for multiparous cows. Furthermore, we also evaluated the duration of different stages of parturition. Results indicated that for both groups, the durations followed the trend: early stage > mid stage > last stage. Correspondingly, the number of images per cow was approximately 84 for primiparous cows and 55 for multiparous cows. Interestingly, during the late stage of parturition, both groups had a similar number of images at approximately 10 per cow (Table 3).

### 3.2. Development and Evaluation of a YOLOv8-Based Model for Automatic Detection of Standing and Lying Postures in Dairy Cows During Calving

**Figure 5:**
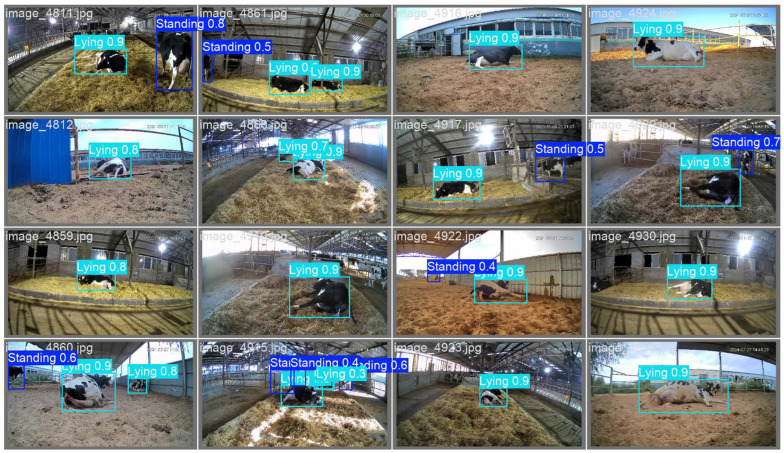
Object detection results of the YOLOv8 model.

Following the preliminary analysis, we developed a YOLOv8 model to automatically detect standing and lying postures of dairy cows during the calving process (Figure 5). Specifically, our results demonstrated that the model achieved higher accuracy in identifying lying postures, effectively distinguishing between standing and lying postures. After completing the training of YOLOv8-based object detection model, we applied the test dataset to evaluate its performance.

**Figure 6:**
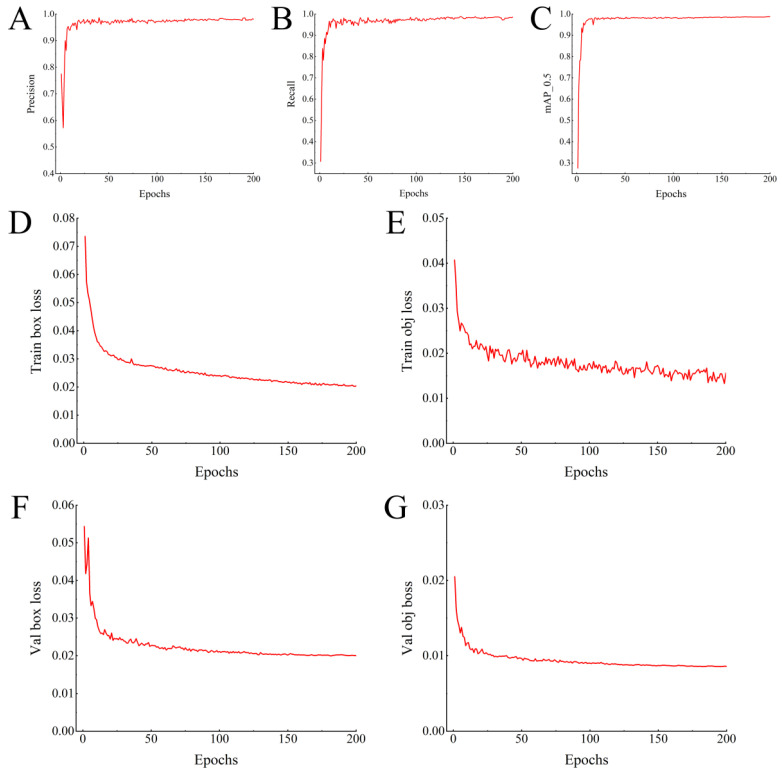
YOLOv8 model performance evaluation. (**A**–**C**) Training performance metrics of the YOLOv8 model. (**D**–**G**) Loss curves during YOLOv8 model training.

During the training phase of the YOLOv8 model, as the number of iterations increased, key performance metrics such as *P*, *R*, m*AP* exhibited an upward trend before stabilizing and converging consistently. At the final stage of training, *P*, *R*, m*AP* reached values of 96.72%, 96.53%, and 97.41%, respectively (Figure 6A–C). The loss curve of YOLOv8 model revealed rapid convergence within the first 25 iterations (Figure 6D–G). Beyond 120 iterations, the loss function values stabilized, indicating that the model had reached its learning boundary and achieved optimal training performance. These results confirm that our YOLOv8 model was successfully constructed.

Following that, images of standing and lying postures from calving cows were input into the trained YOLOv8 model for automatic detection and classification. Performance evaluation metrics were employed to assess its accuracy. The *P* for detecting standing and lying postures was 82.61% and 89.19%, respectively; *R* was 95% and 99%, respectively; m*AP* was 93.82% and 96.35%, respectively; and *F*_1_ scores were calculated as 88.37% for standing postures and 93.84% for lying postures (Table 4).

In order to further validate the accuracy of our model, we conducted comparative analyses under varying lighting conditions and camera angles to evaluate its robustness across diverse scenarios. Remarkably, in independent validation experiments, m*AP* values exceeded 90% across all conditions tested, demonstrating that YOLOv8-based detection model exhibited strong stability and performed as expected in identifying dairy cow postures during calving (Table 5).

### 3.3. Analysis of Postural Behavior Patterns in Primiparous and Multiparous Dairy Cows During Calving

We further analyzed the postural behavior patterns of primiparous and multiparous dairy cows throughout the calving process (Table 6). Concretely, during the entire calving process, the NL was greater than NS. The ratio of NL to NS was 10.31:1 for primiparous cows and 5.43:1 for multiparous cows, with primiparous cows exhibiting 1.71 times more NL than multiparous cows. Notably, both posture types showed a trend of decreasing frequency across the stages of calving, following the order: early stage > mid stage > last stage.

Moreover, the distribution of NL across the early, mid, and last stages was 4.37:2.81:1 for primiparous cows and 2.51:1.55:1 for multiparous cows. Similarly, the distribution of NS across these stages was 9.12:3.80:1 for primiparous cows and 7.00:1.45:1 for multiparous cows. In terms of NPC, primiparous cows exhibited more frequent posture transitions (9.07 changes per cow) compared to multiparous cows (5.29 changes per cow). Furthermore, NPC was the highest during the early stage of calving.

### 3.4. Analysis of Behavioral Changes in Primiparous and Multiparous Dairy Cows During Calving

**Figure 7:**
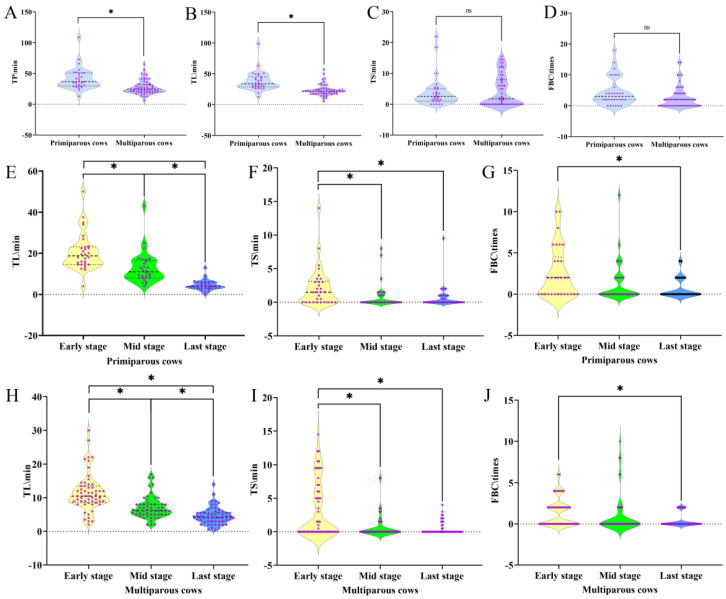
Comparative analysis of calving behavior durations in primiparous and multiparous dairy cows. (**A**–**D**) Differences in time of parturition (TP), time of lying (TL), time of standing (TS), and frequency of behavioral changes (FBC) between primiparous and multiparous dairy cows. (**E**–**G**) Variations in TL, TS, and FBC across different stages of calving in primiparous cows, and (**H**–**J**) corresponding variations in multiparous cows. Note: The * at the top of the graph indicates a significant difference within the same group (*p* < 0.05), while “ns” denotes a non-significant difference within the group (*p* > 0.05). “Early stage”, “Mid stage”, and “Last stage” represent the different phases of the calving process.

In addition to the aforementioned analyses, we investigated the behavioral changes in primiparous and multiparous dairy cows during the calving process. The results showcased that TP and TL were significantly higher in primiparous cows compared to multiparous cows (*p* < 0.05), while TS was notably lower in primiparous cows (Figure 7A–C). FBC was also observed to be higher in primiparous cows than in their multiparous counterparts (Figure 7D). Moreover, among all 86 calving cows included in the study, the maximum TP (109 min) and minimum TP (6 min) were observed in primiparous and multiparous cows, respectively. The average TP for primiparous and multiparous cows was 41.9 min and 27.63 min, respectively. These results indicate that primiparous dairy cows experience a longer calving duration, with lying behavior being the primary activity observed during the calving process.

The duration of calving in dairy cows shows considerable variation across parities and individuals, accompanied by distinct differences in behavioral dynamics during the process. We assessed the behaviors of primiparous and multiparous dairy cows at different stages of calving. The results revealed that TP, TL, TS, and FBC followed a consistent trend across both groups: early stage > mid stage > last stage. Notably, primiparous cows exhibited markedly elevated TL, TS, and FBC during the early stage (*p* < 0.05) (Figure 7E–G), with multiparous cows showing a similar pattern (Figure 7H–J). These findings underscore that standing behavior is most prominent during early-stage calving. Furthermore, the FBC of primiparous cows was 1.72 times higher than that of multiparous cows, indicating a greater frequency of lying-to-standing transitions in primiparous cows, particularly in the early stage compared to subsequent stages.

### 3.5. Correlation Between Calf Birth Weight and Maternal Behavioral Patterns During Parturition in Primiparous and Multiparous Dairy Cows

**Figure 8:**
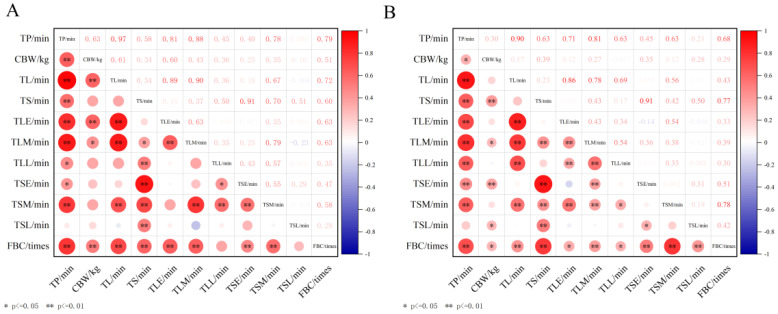
Comparative analysis of calving behavior durations in (**A**) primiparous and (**B**) multiparous dairy cows. TP: time of parturition (min), CBW: calf birth weight (kg), TL: time of lying (min), TS: time of standing (min), TLE: time of lying during early stage (min), TLM: time of lying during mid stage (min), TLL: time of lying during last stage (min), TSE: time of standing during early stage (min), TSM: time of standing during mid stage (min), TSL: time of standing during last stage (min), FBC: frequency of behavior change (time).

Ultimately, we calculated duration metrics associated with the calving process and performed Pearson correlation analysis to explore relationships among various behavioral indices in dairy cows during parturition. In primiparous cows, calf birth weight was significantly positively correlated with several behavioral metrics: TP (*p* < 0.01, r = 0.63), TL (*p* < 0.01, r = 0.61), time spent lying during the early stage (*p* < 0.01, r = 0.60) and mid stage (*p* < 0.05, r = 0.43), as well as FBC (*p* < 0.01, r = 0.51) (Figure 8A). In multiparous cows, positive correlations were observed between calf birth weight and TP (*p* < 0.05, r = 0.30), TS (*p* < 0.01, r = 0.39), time spent lying during the mid stage (*p* < 0.05, r = 0.27), time spent standing during the early stage (*p* < 0.01, r = 0.35), time spent standing during last stage (*p* < 0.05, r = 0.28), and FBC (*p* < 0.05, r = 0.29) (Figure 8B). These results indicate that calf birth weight is strongly linked to maternal behavioral patterns during labor—particularly the duration and frequency of specific behaviors such as lying or standing at different stages of parturition—and highlight their potential as indicators for monitoring calving progress and predicting outcomes. For primiparous cows specifically, stronger correlations were observed between calf birth weight and lying-related behaviors or FBC compared to multiparous cows where correlations were more evenly distributed across a wider range of behaviors. It is important to note that while calf birth weight is a key parameter of neonatal health and vitality that may reflect maternal physiological adaptations and behavioral responses during labor, correlation does not necessarily indicate causation.

## 4. Discussion

With the continuous development of large-scale dairy farms, traditional manual monitoring methods in the dairy cow parturition process have increasingly revealed issues such as time consumption, labor intensity, and low accuracy. Therefore, intelligent monitoring systems based on computer vision technology and deep learning algorithms have become crucial for addressing these problems. In recent years, machine vision technology, particularly YOLO-based models, has been widely applied in dairy cow behavior recognition and object detection [11,20,23]. This study leveraged YOLOv8 model for accurate identification of key behavioral patterns during bovine parturition. A dataset of 600 videos, yielding 10,544 image samples through frame-by-frame processing (total duration: 3085 min), was used to build a calving posture recognition model. Subsequently, complete video recordings of 86 parturient cows (30 primiparous and 56 multiparous) were analyzed to investigate changes in calving behavior. In conclusion, our study offers a scientific basis and technological support for parturition monitoring in dairy cows, assisting farm managers in timely interventions. This could effectively reduce the risk of dystocia, enhance dairy cow health management, and promote intelligent farm management.

In our study, real-time monitoring with computer vision and the YOLOv8 model significantly improves the accuracy of parturition behavior recognition, with *P*, *R*, and m*AP* reaching 96.72%, 96.53%, and 97.41%, respectively. The automatic detection of standing and lying postures yielded consistent results and high precision. For the test dataset, the *P* for detecting lying postures was 89.19%, and standing postures achieved 82.61%. Notably, the *R* and *F*_1_ scores for lying postures were higher, with values of 99% and 93.84%, respectively. This difference may be due to the more distinct and easily identifiable nature of lying postures compared to standing postures. Moreover, the model’s robustness was further validated through independent validation experiments under varying lighting conditions and camera angles. Undoubtedly, light intensity and duration can also affect calving, particularly during recordings under natural daylight and nighttime auxiliary lighting conditions. Previous studies have highlighted the impact of lighting on calving behavior [24]. Specifically, research has shown that cows exposed to 16–16.25 h of light per day produce higher milk yields than those under natural light conditions [25]. Proper lighting can improve dairy cow performance and may indirectly influence calving by enhancing overall health and behavior. Moreover, extended lighting duration can increase feed intake and overall production efficiency, which may also affect calving [26]. Unfortunately, due to the constraints of our study, conducted on a commercial farm, and to avoid impacting the farm’s economic efficiency, we did not conduct further analysis on this aspect.

Our results revealed that the YOLOv8 model maintained high stability, with m*AP* values exceeding 90% across all tested conditions, further confirming its reliability and effectiveness in real-world scenarios. Furthermore, when comparing the calving processes of primiparous and multiparous cows, we observed significant differences in the duration of each stage. The calving process of primiparous cows was slower, particularly during the early stages of parturition. There were also differences in the postural behavior of the cows. Primiparous cows exhibited a higher proportion of standing postures, while multiparous cows spent more time in lying postures. This difference may be attributed to the physiological differences between primiparous and multiparous cows during calving [27,28], suggesting that monitoring strategies should be tailored to the specific physiological states of the cows.

Calving is a distressing process accompanied by acute pain [29]. In this study, we analyzed the postural behavior patterns of primiparous and multiparous dairy cows during the calving process, revealing significant differences in posture transitions between the two groups. Our findings indicate that primiparous cows exhibited a significantly higher NL compared to NS, with the ratio of NL to NS being 10.31:1 for primiparous cows and 5.43:1 for multiparous cows. This suggests that primiparous cows, due to their physiological and behavioral characteristics, tend to assume lying positions more frequently than multiparous cows [30]. The higher frequency of lying in primiparous cows may be related to their lack of experience and weaker adaptation to the calving process compared to multiparous cows. Moreover, both posture types showed a decreasing frequency as the calving process progressed, with the highest frequency observed in the early stage and the lowest in the last stage of calving. The distribution of lying behavior in the early, mid, and last stages for primiparous cows was 4.37:2.81:1, while for multiparous cows it was 2.51:1.55:1. In contrast, the distribution of standing behavior across these stages was 9.12:3.80:1 for primiparous cows and 7.00:1.45:1 for multiparous cows. This indicates that primiparous cows maintain a more stable frequency of standing posture throughout the calving process, while multiparous cows exhibit greater fluctuations, likely due to differences in their physiological stress and behavioral patterns during calving [31]. Regarding the NPC, primiparous cows exhibited more frequent posture transitions (9.07 changes per cow) compared to multiparous cows (5.29 changes per cow), suggesting that primiparous cows adjust their behavior more frequently during calving. This may be attributed to the lack of experience and their need to adjust to the varying physical demands of the calving process [28]. Notably, the highest frequency of posture changes was observed during the early stage of calving, which could be due to the more pronounced adaptive behavior required for primiparous cows during the initial stages of labor [30].

In terms of time, our results show that primiparous cows had significantly higher TP and TL compared to multiparous cows (*p* < 0.05), while their TS was notably lower. Furthermore, the FBC was also higher in primiparous cows than in multiparous cows. These findings suggest that primiparous cows experience longer calving durations, with lying behavior being the primary activity observed during the calving process [32,33,34]. Behavioral dynamics during calving show considerable variability across different parities and individuals. We assessed the behaviors of both primiparous and multiparous cows at different stages of calving, and found that TP, TL, TS, and FBC followed a consistent trend across both groups: early stage > mid stage > last stage. Notably, primiparous cows exhibited significantly higher TL, TS, and FBC during the early stage (*p* < 0.05), with multiparous cows showing a similar pattern. These findings emphasize that standing behavior is most prominent during the early stages of calving [33,35]. Moreover, the FBC of primiparous cows was 1.72 times higher than that of multiparous cows, indicating that primiparous cows had a higher frequency of lying-to-standing transitions, especially during the early stage of calving [36]. These results further highlight the behavioral differences between primiparous and multiparous cows during the calving process. Primiparous cows exhibit more frequent lying and standing transitions during early-stage calving, which is likely linked to their higher physiological stress and greater need for behavioral adjustments during labor. The application of machine vision technology to monitor postural changes and behavioral dynamics provides accurate data for managing cows during calving, offering valuable insights for improving calving management and cow welfare.

Maternal health status and fetal weight are key factors influencing calving behavior in dairy cows [37]. This study calculated duration metrics associated with the calving process and performed Pearson correlation analysis to explore the relationships between maternal behavioral patterns and calf birth weight during parturition in primiparous and multiparous dairy cows. Calf birth weight is a critical indicator of neonatal health and vitality, reflecting maternal physiological adaptations and behavioral responses during labor. Our results revealed that, in primiparous cows, calf birth weight was significantly positively correlated with several behavioral metrics: TP (*p* < 0.01, r = 0.63), TL (*p* < 0.01, r = 0.61), time spent lying during the early and mid stages of calving (*p* < 0.01, r = 0.60; *p* < 0.05, r = 0.43), and FBC (*p* < 0.01, r = 0.51) (Figure 8A). In multiparous cows, positive correlations were observed between calf birth weight and TP (*p* < 0.05, r = 0.30), TS (*p* < 0.01, r = 0.39), time spent lying during the mid stage of calving (*p* < 0.05, r = 0.27), time spent standing during the early stage (*p* < 0.01, r = 0.35), time spent standing during the last stage (*p* < 0.05, r = 0.28), and FBC (*p* < 0.05, r = 0.29). These findings suggest that calf birth weight is strongly correlated with maternal behavioral patterns during labor [38], particularly the duration and frequency of behaviors such as lying or standing at different stages of parturition. These behavioral metrics could serve as potential indicators for monitoring the progress of calving and predicting outcomes. For primiparous cows, stronger correlations were observed between calf birth weight and lying-related behaviors or FBC, whereas in multiparous cows, the correlations were more evenly distributed across a broader range of behaviors. Specifically, primiparous cows may exhibit greater physiological stress and behavioral adjustments during parturition [39], leading to stronger associations between their behavioral patterns and calf birth weight. On the other hand, multiparous cows, having undergone multiple calvings, likely demonstrate more adaptive behaviors [30], resulting in a more uniform distribution of correlations between calf birth weight and a variety of behaviors. This finding provides new insights into the mechanisms underlying maternal behavioral changes during calving and highlights the potential of these behaviors for predicting calving outcomes.

Nonetheless, this study has several limitations. The sample size may not fully represent the heterogeneity of dairy farming systems, particularly those with varying breeds, environments or management practices, potentially limiting the generalizability of the findings. In addition, the impact of dystocia on behavioral patterns or calving time is an area that warrants further exploration. As mentioned earlier, the specific timing and intensity of lighting, as well as the color of the lighting equipment, may also influence calving time—especially in our study, which was conducted under natural daylight during the day and auxiliary lighting at night. Furthermore, while this study primarily focused on posture recognition, additional research is needed to investigate the underlying causes and physiological states driving these behaviors to refine management strategies. Finally, despite the model’s capacity for real-time monitoring, practical implementation in commercial farms may encounter challenges such as hardware limitations, computational capacity, and cost-effectiveness.

## 5. Conclusions

In conclusion, this study demonstrates the effectiveness of making use of YOLOv8 model for real-time, non-contact recognition of calving postures and behaviors in dairy cows. The model achieved high accuracy, with *P*, *R*, and m*AP* values of 96.72%, 96.53%, and 97.41%, respectively. Behavioral analysis revealed significant differences between primiparous and multiparous cows in terms of posture transitions and the duration of various behaviors. The proposed model offers an efficient tool for monitoring the calving process and provides valuable insights for intelligent farm management. 

## Figures and Tables

**Table 1:** Behavioral postures of cows during the calving process.

Cow Behavior During Calving	Description	Number of Images
Standing	The cow stands upright with all four legs on the ground	4548
Lying	Part of the cow’s body is in contact with the ground	5996

**Table 2:** Experimental environment and equipment.

Category	Name	Details
Hardware Configuration	GPU model	NVIDIA GeForce RTX 4080 (NVIDIA, Santa Clara, CA, USA)
	CPU model	Intel Core i9-13900HX (Intel, Santa Clara, CA, USA)
	Memory capacity	64G DDR4 (Samsung, Seoul, Republic of Korea)
Software Configuration	Python version	3.9.0
	Pytorch version	2.3.1
	CUDA	11.8

**Table 3:** Comparison of calving data between primiparous and multiparous cows.

Group	Number of Images	Calf Birth Weight (kg/head)	Total Calving Process Video Duration (min)				Total Calving Process Images (Image)			
			Total	Early Stage	Mid Stage	Last Stage	Total	Early Stage	Mid Stage	Last Stage
Primiparous cows	30	37.32	1257	687.5	416.00	153.5	2514	1375	832	307
Multiparous cows	56	38.96	1547	825.8	437.5	284	3094	1651	875	568

**Table 4:** Performance evaluation of the YOLOv8 model for detecting standing and lying postures in calving cows.

Label	*P*/%	*R*/%	m*AP*/%	*F*_1_ Score/%
Lying	89.19	99	96.35	93.84
Standing	82.61	95	93.82	88.37

Note: *P* represents precision; *R* signifies recall; and m*AP* stands for mean average precision.

**Table 5:** Robustness validation of YOLOv8 model for dairy cow posture detection under diverse conditions.

Lable	Lighting Condition			Camera Angle		
	Daytime	Nighttime	Rainy	Head	Tail	Abdomen
Lying (m*AP*)	96.3%	94.3%	96.3%	94.5%	91.1%	99.5%
Standing (m*AP*)	92.5%	91.4%	90.4%	90.2%	90.2%	99.3%

Note: m*AP* represents mean average precision.

**Table 6:** Postural behavior patterns of primiparous and multiparous dairy cows throughout the calving process.

Group	Posture	Total	Early Stage	Mid Stage	Last Stage
Primiparous cows (*n* = 30)	NL (images per cow)	79.71	42.47	27.46	9.77
	NS (images per cow)	7.73	4.47	1.86	0.49
	NPC (occurrences per cow)	9.07	5.20	2.67	1.33
Multiparous cows (*n* = 56)	NL (images per cow)	46.66	23.13	14.30	9.23
	NS (images per cow)	8.59	6.36	1.32	0.91
	NPC (occurrences per cow)	5.29	2.79	1.79	0.71

Note: NL indicates the number of lying events; NS represents the number of standing events; and NPC refers to number of posture changes.

## Data Availability

The original contributions presented in this study are included in the article. Further inquiries can be directed to the corresponding author.
